# mSphere of Influence: Rapid evolution of pathogenesis and drug resistance in human pathogenic fungi

**DOI:** 10.1128/msphere.00570-24

**Published:** 2025-02-25

**Authors:** Pengjie Hu

**Affiliations:** 1 Institute of Microbiology, Chinese Academy of Sciences 85387 , Beijing, China; Shenzhen Institute of Synthetic Biology, Chinese Academy of Sciences, Shenzhen, China

**Keywords:** human fungal pathogen, evolution, genetic mutation, pathogenesis, drug resistance, adaptation

## Abstract

Pengjie Hu works in the field of fungal pathogenesis, drug resistance, and evolution. In this mSphere of Influence article, he reflects on how three works, “Transposon mobilization in the human fungal pathogen *Cryptococcus* is mutagenic during infection and promotes drug resistance in vitro” and “Genome-wide analysis of heat stress-stimulated transposon mobility in the human fungal pathogen *Cryptococcus deneoformans*” by Gusa et al. and “Rapid evolution of an adaptive multicellular morphology of *Candida auris* during systemic infection” by Bing et al. have impacted his work on the evolution of virulence, resistance, and adaptation in human fungal pathogens.

## COMMENTARY

Invasive human fungal pathogens are a major cause of infectious morbidity and mortality, particularly in immunocompromised individuals, and they result in approximately 3.75 million deaths annually (1, 2). A major reason for the enormous damage caused by fungal infections is the limited number of antifungal drugs available for treatment (3, 4). In addition, many fungi are capable of undergoing genomic mutations, evolving into more adaptable forms, and even developing multi-drug resistance. During the infection process, fungi face various stresses, including those posed by host immune defenses and by antifungal drugs. The genetic mechanisms that allow some fungi to rapidly adapt to these stresses are, thus, strongly related to persistence within the host and to the development of fungal virulence and drug resistance. The genetic mutations underlying these changes potentially involve base substitutions, deletions, copy number variations, and chromosomal duplications, but the movement of mobile DNA elements (transposons) within the genome also can alter the genetic landscape. The works “Transposon mobilization in the human fungal pathogen *Cryptococcus* is mutagenic during infection and promotes drug resistance *in vitro*” and “Genome-wide analysis of heat stress-stimulated transposon mobility in the human fungal pathogen *Cryptococcus deneoformans*” by Gusa et al. (5, 6) and “Rapid evolution of an adaptive multicellular morphology of *Candida auris* during systemic infection” by Bing et al. (7) provide comprehensive insights into the evolution of fungal genomes that facilitates the formation of drug-resistant and hyper-virulent strains during infection.

The work of Gusa et al. (5, 6) provides a comprehensive genome-wide evaluation of heat stress-induced transposon mobilization in the human fungal pathogen *C. deneoformans*. The authors found that transposon copies accumulated more rapidly in genomes of fungi grown at host body temperatures, and multiple transposable elements were identified in the genomes of *Cryptococcus* isolated from infected mice. They also determined that the majority of mutations conferring drug resistance were due to gene inactivation by transposon elements. These results suggest that transposon mobilization activated by temperature stress in the host, or potentially in the environment, is a previously uncharacterized adaptative mechanism that contributes to the enhancement of virulence of *Cryptococcus* and the acquiring of drug resistance (5, 6). In addition to transposable element-mediated genome evolution, Bing et al. (7) found that more localized genetic mutations, specifically *de novo* point mutations, can trigger the rapid evolution of *C. auris* to an adaptive multicellular morphology during systemic infection. The authors used *in vivo* imaging and genome sequencing to analyze morphological and genetic characteristics of *C. auris* isolated from a patient with a bloodstream infection. They found that the fungus had evolved into a multicellular form with filamentous growth, which increased its virulence and ability to persist in the host. This morphological switch was accompanied by point mutations that induced changes to the expression of genes related to filamentous growth and cell wall integrity. These findings provide insight into mechanisms underlying the rapid evolution of *C. auris* during infection and thus its ability to adapt to host conditions. The evolution of multicellular morphology may play a key role in the pathogenesis of *C. auris* by allowing the fungus to evade host immune defenses and establish systemic infections (7).

These studies have significantly impacted my understanding of fungal adaptation and evolution. The work of Gusa et al. (5, 6) indicated that transposons are not merely “junk DNA” but rather dynamic elements that can rapidly reshape fungal genomes in response to environmental cues. The temperature-dependent nature of transposon mobility highlights the exquisite sensitivity of fungal genomes to environmental stresses and underscores the importance of studying fungal evolution in ecologically relevant contexts. The work by Bing et al. showed that host stress can cause transposon-independent fungal mutations to accumulate. These mutations can alter gene expression leading to morphological switches that facilitate adaptation to stressful *in vivo* conditions. In support of this finding, our recent work identified a previously undescribed human fungal pathogen, *Rhodosporidiobolus fluvialis* (8). Many species in the genus *Rhodosporidiobolus*, including *R. fluvialis*, are highly resistant to fluconazole and caspofungin. Importantly, in this new fungal pathogen, mammalian body temperature induces mutagenesis, which efficiently drives the development of hypervirulence and pan-drug resistance (8). Fungal pathogens and other microbes often face multiple challenges to their survival, such as nutrient scarcity, antibiotics, changes in physical and chemical conditions, and predators or host immune factors. A key principle that microbial populations adhere to as they adapt to complex and ever-changing environments is to “not put all their eggs in one basket.” When confronted with survival challenges, different subpopulations of cells may adopt different coping strategies. For example, under antibiotic exposure, some cells may enter a low-metabolic dormant state, enhancing their resistance to antibiotics, and under appropriate conditions, certain cells of *C. deneoformans* can transition from yeast to hyphal forms, facilitating nutrient acquisition or resistance to phagocytosis by amoebae. The work of Gusa and Bing et al. has demonstrated that, in addition to altering cellular physiological metabolism or developmental states, cells can take advantage of modifications of genetic information to survive under stressful conditions. Although mutation is the fundamental genetic driver of evolution, our understanding of how the environment influences mutation rate and spectrum remains limited. Interestingly, the previous study has found that fungal mutation rates vary significantly under different environmental conditions, suggesting that various environmental factors may play a role in driving genetic mutations in fungi (9).

These findings raise a fundamental question: how do cells within a microbial population generate these heterogeneous behaviors, and how are these apparent decisions made? Within a cellular population, individual cells may differ in their perceptions of and responses to environmental stimuli, ultimately leading them to employ different strategies to cope with survival pressures. One question I am particularly interested in is whether it is possible to predict cell fate selection at the single-cell level, and, in particular, whether we can predict which cells are capable of undergoing genetic mutations or epigenetic changes. Answering these questions relies on breakthroughs in technological approaches, such as single-cell sequencing, real-time super-resolution microscopy, and the integration of multi-omics data. The work of Gusa and Bing et al. suggests that mutagenesis in response to stresses during infection likely contributes to phenotypic variation that promotes adaptation, survival, and virulence, as well as the development of antifungal-drug resistance. It will be important to identify the specific environmental triggers and molecular mechanisms that drive such genetic mutations, as this information promises to provide deep insights into the complex interplay among environmental stresses, genetic mutations, and fungal adaptation and to assist the development of novel treatments for fungal infections. The findings have also sparked a broader conversation about the potential impact of climate change on the prevalence and virulence of fungal pathogens (10, 11), highlighting the need for increased surveillance and research in this area.
